# COVID-19: the perfect vector for a mental health epidemic

**DOI:** 10.1192/bjb.2020.60

**Published:** 2020-06-01

**Authors:** Idura N. Hisham, Giles Townsend, Steve Gillard, Brishti Debnath, Jacqueline Sin

**Affiliations:** 1 St George's Hospital Medical School, UK; 2 Surrey and Borders Partnership NHS Foundation Trust, UK; 3 St George's, University of London, UK; 4 University of Reading, UK

**Keywords:** COVID-19, pandemic, epidemic, mental health, isolation, social distancing, public health, abuse, stigma

## Abstract

In times of crisis, people have historically had to band together to overcome. What happens when they cannot? This article examines the reality of people forced to isolate from one another during one of the most turbulent events of their lives: the COVID-19 pandemic. Connecting the dots of topics including fear, social stigmas, global public response and previous disease outbreaks, this article discusses the negative mental health effects that individuals and communities will likely suffer as the result of social distancing, isolation and physical infection.

## The rise of a new pandemic

On 31 December 2019, the Chinese authorities reported a disease that had appeared in the Hubei province to the World Health Organization (WHO) as a ‘pneumonia of unknown aetiology.’^1^ That ‘pneumonia’ is now known as the novel coronavirus disease (COVID-19). As of 13 May 2020, there are 4 170 424 confirmed cases of COVID-19 with 287 399 deaths reported globally, and these numbers are continuing to grow.^2^

While current strategies to fight the outbreak primarily focus on curbing the spread and treating the infected, it is crucial to consider the effects of COVID-19 on the wider population's mental health, in the short, medium, and long term. By studying past new emerging infections (NEIs), in particular, severe acute respiratory syndrome (SARS) in 2003, Middle East respiratory syndrome (MERS) in 2012 and 2015, and H1N1 in 2009 (the only one to also be declared a pandemic),^3^ we can better understand, potentially predict, and thus counteract the possible effects of COVID-19 on mental health.

The 2003 SARS epidemic is one such case study of how infectious disease outbreaks affect mental health, with this particular epidemic described as a mental health catastrophe.^4^

Hong Kong was disproportionately affected in the SARS epidemic, with up to 1755 individuals infected and 299 deaths.^4–7^ A study conducted soon after the outbreak indicated that a significant proportion of the Hong Kong population, including those not infected with the disease, displayed moderate to severe psychiatric symptoms, meeting diagnostic thresholds of common mental disorders such as depression and generalised anxiety disorder.^4^ These effects are not specific to SARS, but are a feature seen in most, if not all, infectious disease outbreaks. A study of a hospital in South Korea found that 70% of MERS patients admitted to hospital presented with a psychiatric symptom, and 40% of them were later prescribed medication to alleviate the symptoms.^8^ In both SARS and MERS, the psychiatric implications continued far beyond the outbreak, with many having persistent mental health issues years afterwards.^4,8–13^ The same effects, albeit of varying ferocity, could also be seen during the H1N1 outbreak.

COVID-19 is of a scale that the current generation has never seen before, with the ‘Spanish flu’ of 1918 potentially being the last outbreak to have had such widespread effects. However, owing to the scarcity of literature evidencing the mental health effects of the Spanish flu pandemic, and the time-gap of more than a century, in which our society, health and financial systems have all changed beyond our forefathers’ imagination, limited parallels can be drawn between current and older pandemics other than mortality. Drawing parallels with SARS and MERS also has its limitations. Studies of SARS patients have varying degrees of reliability owing to inconsistent study design, research methods, and standardised measures being used across the different studies – a common problem with research done in the early aftermath of a disaster.^11^ The existing literature surrounding SARS and MERS is also primarily focused on Asian countries, as they were most affected by the outbreaks; this potentially limits its generalisability to Western countries, which have a more ‘individualistic’ structure compared with the ‘collectivist’ societal systems of those nations. To minimise this limitation, our focus was to identify and learn from themes that recur in different disease outbreak settings. Given that the COVID-19 pandemic is already more global and longer lasting than any outbreaks we have faced in recent memory, one may extrapolate that its mental health implications will be at least as severe as those of others NEIs. We provide a brief overview of the potential negative ramifications in store if mental health is not given more priority in the current outbreak response.

## Why do NEIs contribute to increases in mental health issues?

Throughout history, the emergence and increasing prevalence of infectious agents have coincided with an increased risk of psychiatric manifestations. NEIs such as SARS and COVID-19 adversely affect mental health in a multitude of ways, permeating at individual, communal and societal levels. The most common psychological morbidities include worries, anxiety, mood disturbances, poor sleep and hypochondriac beliefs.^14–17^ Pervasive feelings of hopelessness, uncertainty and fear tend to dominate society during such outbreaks, as a result of life as we know it stopping or changing.^13,15–18^ Such feelings may be born out of an increased perceived threat, which drives ‘safety’ behaviours in individuals and community that can be maladaptive.^19^ The most common behaviours of this nature include hypervigilance (i.e. looking out for potential dangers) and avoidance (i.e. keeping ourselves from sources of danger or threat).^19^ Intense fear and panic are also used as excuses, albeit often unintentionally, for unjustified discriminatory behaviour such as xenophobia and stigmatisation of particular groups, or patterns of hoarding supplies.^20^

## Fear

‘*This is a time for facts, not fear. This is the time for science, not rumours. This is the time for solidarity, not stigma*,’^21^ said Tedros Adhanom, the Director-General of the WHO, in reference to COVID-19 on 12 February 2020.

Fear was preponderate in affected populations (including healthcare workers) during SARS: not only for personal safety but for the safety of others. At the time, SARS was unique in its psychosocial effects, evoking a deep-rooted fear of infecting family and community members.^7,11,12^ In Hong Kong, the government's perceived lack of control in containing the SARS outbreak led to a pervasive sense of hopelessness in the citizenry, a psycho-emotional factor amplified and perpetuated by the media. This, in turn, led to general apprehension and panic.^22^ The influence of the ‘rumour mill’ during an outbreak must be taken seriously; as the desire for facts escalates, any absence of clear and accurate messaging can augment popular anxiety, driving people to seek information from less reliable sources. This same trait is now evident in the context of COVID-19, exacerbated by media and popular discourse promulgating paranoia and anxiety.^23,24^

Social media has an important role in shaping the public's risk perception;^24^ however, it can also be a vessel for the fast dispersal of false news, which can bring with it disastrous consequences. During the H1N1 pandemic, widespread misinformation surrounding the vaccine has been implicated in reduced uptake and increased hesitancy.^25,26^ The current COVID-19 outbreak has seen a repeat of this, with the spread of ‘fake news’ through social media contributing to significant misinformation, leading to fear, panic and even non-compliance with infection control measures. The influence of social media in propagating misinformation during COVID-19 has even led to protests against lockdown measures in the UK with protestors chanting phrases such as ‘Stop 5G!’ – referring to a theory made popular through social media.^27^ This influence has persisted despite the UK government forming a rapid response unit to tackle issues on misinformation early in the outbreak response.^28^

Fear can be beneficial to a point during an outbreak, leading to behaviours which reduce the spread of the disease. Excessive fear, however, can lead to irrational beliefs that impede infection control measures and can probably precipitate maladaptive coping techniques, albeit unintentionally.^29,30^ A survey showed that 66% of young adults in the UK avoided news on COVID-19 as it was unhelpful for their mental health.^31^ This highlights how, although fear is an important tool in public health messaging, excessive fear can not only impede its reach but also potentially exacerbate a different public health issue.

## Stigma

Stigma was also linked to mental health morbidity in the SARS outbreak.^32^ This included self-stigmatisation (individuals continuing to feel ‘polluted’ or ‘contaminated’ up to 16 months after the outbreak), professional stigmatisation (denigration of healthcare workers and figures of authority) and, of course, racial stigmatisation (people of Asian descent being painted as social pariahs).^11,12,32^ In another parallel with the SARS and MERS outbreaks, the COVID-19 pandemic has spurred racial stigmatisation, especially toward those of Chinese heritage, in the form of xenophobia and discrimination.^33–35^ A systematic review identified that the perception of having been a victim of stigmatisation due to SARS was one of the most consistent aetiological factors for the development of psychiatric disorders and chronic fatigue syndrome.^11^ Therefore, preventing stigmatisation during COVID-19 should be made a priority in order to prevent similar adverse outcomes in COVID-19 patients and in the wider population.

Stigma not only affects the mental health of individuals, it can also disrupt infection control measures. Barrett and Brown^36^ identified four elements of stigma that can contribute to this.

• Stigma can present major barriers against healthcare-seeking, thereby reducing early detection and treatment and furthering the spread of disease.
• Social marginalisation often can lead to poverty and neglect, thereby increasing the susceptibility of certain groups to infectious diseases.
• Potentially stigmatised populations may distrust health authorities and resist cooperation during a public health emergency.
• Social stigma may distort public perceptions of risk, resulting in mass panic among communities and the disproportionate allocation of healthcare resources by politicians and health professionals.

Stigmatisation and discrimination have socioeconomic ramifications within populations, as well as being related to feelings of fear, creating a destructive, mutually reinforcing dynamic.^32^

## Quarantine and social isolation

The negative influences of quarantine and isolation on mental health have been described at length.^23,37^ Adverse effects on mental health often persist for months after the end of isolation, and those with pre-existing mental health conditions are at higher risk of prolonged adverse effects, as shown by both the SARS and MERS outbreaks.^13,38,39^ Discrimination, social shunning, violence and vandalism of property are among the consequences of the maltreatment faced by quarantined people at the hands of others in society.^23^

Most adverse effects from quarantine stem from restricted liberties, whereas voluntary quarantine is associated with less distress and fewer long-term complications.^37^ Earlier in the pandemic response, the UK relied on the altruistic nature of the public to practice ‘social distancing’, but as of 23 March 2020, police have had the authority to enforce this through fines and other penalties. According to a recently published report, the specific concerns of the UK population in regards to isolation measures included having to separate from others in the household (45%), getting supplies (41%), mental health implications (37%), social life implications (24%), loss of income (22%) and finding someone to cover caring responsibilities (12%). In addition, those between 18 and 34 years old were more likely to report negative mental health effects.^40^

The economic sequalae of COVID-19 lockdown measures in the UK have led to businesses closing and many losing employment; the Bank of England has warned that unemployment rates could rise to 9% (compared with 4% earlier this year).^41^ Increased unemployment poses significant public health risks. For instance, in 1981, when unemployment rates in the UK increased by 3.6%, suicide rates also increased by 2.7%.^42^ Reports from the 2008 recession echoed this and showed that the resultant mass unemployment was associated with a 4.45% increase in suicide rates in 26 European Union countries.^42^ Although the end of lockdown is expected to improve the economic downturn, many that have lost their jobs will struggle to find new employment as companies reduce hiring,^41^ further protracting the financial and psychological effects of COVID-19.

Quarantine and isolation are necessary measures and, as of now, appear to be among the most effective means of containing the outbreak.^43,44^ With the possibility of mass quarantine measures having to be reimplemented owing to ‘second waves’ of COVID-19, as seen in several countries,^45–47^ the concerns of the public must be addressed to mitigate the negative effects of this potentially recurring ‘necessary evil’.

## Loss of protective factors

Rutter defined protective factors as those that ‘modify, ameliorate or alter a person's response to some environmental hazard that predisposes to a maladaptive outcome’.^48^ Protective factors may exist in individuals or in the family, and in institutional or community contexts. They can also be biological or psychosocial in nature.^49^ In times of duress, social support is one of the protective factors against the development of mental health disorders such as depression and post-traumatic stress disorder (PTSD).^50,51^ Nevertheless, social distancing is a necessary public health response to NEIs. In the UK, people are now prohibited from both large and small gatherings with those from different households. This has, for example, led to religious institutions cancelling services, which ordinarily constitute a major source of support, particularly for the elderly.^52^

Social support is just one of many examples of a lost protective factor resulting from COVID-19. The public also has to face financial instability, unemployment and disrupted routine.

Pandemics and epidemics not only increase the many risk factors for mental health morbidities but also pull away protective factors simultaneously; these effects compound one another.

## Increased risk of abuse

Reports have already emerged of increased cases of domestic abuse among the populations affected by COVID-19, with a UK abuse charity, Refuge, seeing a 700% increase in traffic to their hotline website in a day.^53^ It is important to note that domestic abuse is not always physical – it can also be psychological, financial or sexual. Not only can COVID-19 exacerbate existing cases of abuse, the stress associated with it can also lead to new cases. Social isolation can mean spending significantly more time at home with abusive family members, with no escape or respite.^54^ Furthermore, a pandemic increases financial and psychological stresses, which are associated with increased likelihood of abusive behavior.^55^

The significant risk of abuse towards the elderly should not be overlooked. A study carried out by Reay and Browne in 2001 identified 15 risk factors in caregivers that increase the risk of mistreatment. Three of them are particularly relevant during the current outbreak: (a) caregivers who are subject to high stress and strain; (b) those who live with elderly patients; and (c) those who are isolated and lack community and personal support.^56^ Furthermore, feelings of anxiety in caregivers are also associated with neglect.^56^ For the elderly who require greater assistance with daily activities, as well as those with dementia, caregiver stress is a predominant factor in the onset of abuse.^57^ COVID-19 intensifies all these risk factors in caregivers, thus placing the elderly at a higher risk of abuse or neglect. Although the UK government has already issued measures to address abuse,^58^ there remains a question of how accessible and practical these technology-driven measures are for the elderly population.

Pandemics such as COVID-19 may also make it more difficult for victims to receive help, owing to its influence on an already overwhelmed public health infrastructure,^59^ including effects on the social care system, reduced philanthropic donations to abuse charities and imposed travel limitations.^54^ Involvement in abuse, either as a perpetrator or a victim, exerts an enduring effect on both physical and mental health.^60^ The stress factors associated with COVID-19, if not properly mitigated, will make the current pandemic an ideal environment for abuse to thrive, with lifelong, adverse effects on the health of those involved.

## COVID-19, PTSD and intensive treatment

Approximately one in five critically ill patients and their partners will develop clinical symptoms of PTSD and reduced reported health-related quality of life as a result of their intensive treatment unit (ITU) stay.^61^ The estimated number of COVID-19 patients requiring intensive care owing to, for instance, acute respiratory distress syndrome (ARDS) currently stands at about 15–30%.^62^ Patients admitted to ITUs, as well as their families, are at risk of developing post intensive care syndrome (PICS) – a physical, cognitive and mental disorder associated with an ITU stay. The mental health impairments that can arise among these patients include depression, anxiety and PTSD.^63^ Existing mental health conditions also increase the risk of developing PICS, in both patients and their families.^64^

Furthermore, the use of extracorporeal membrane oxygenation (ECMO), also known as extracorporeal life support, in the treatment of COVID-19 poses a specific mental health risk that warrants consideration.^65,66^ ECMO, which supports the lungs and/or the heart, is considered one of the most invasive rescue therapies and has high rates of adverse mental health outcomes (e.g. PTSD) in patients post-treatment. The prevalence of PTSD in patients who were on ECMO is estimated to be between 11 and 27%, at least a four- to five-fold increase from general population prevalence figures.^67,68^ Moreover, compared with other ARDS survivors, those who were on ECMO also reported lower quality of life and lower rates of return to employment.^67^

## Mental health services and COVID-19

The UK government does not currently recognise people with existing mental health conditions as part of the ‘vulnerable population’, because their risk of getting seriously ill from COVID-19 is perceived as low. However, these groups are vulnerable to an exacerbation of pre-existing mental health conditions. Those with pre-existing mental health conditions often suffer greater psychological distress in instances of an adverse event or situation.^69,70^

Moreover, this cohort is often in poorer physical health, with fewer protective factors such as healthy lifestyle or an active social support network, making them physically and mentally vulnerable to the effects of COVID-19. One example is smoking, which is estimated to be twice as prevalent among people with mental disorders, with higher reported mental health disease severity directly correlated with numbers of cigarettes smoked.^71^ In addition, these patients have a higher incidence of chronic infections owing to substance abuse and socioeconomic deprivation.^72^ This is particularly relevant to COVID-19, as those with chronic respiratory illness, such as chronic obstructive pulmonary disease (which is directly correlated with smoking frequency), are at higher risk of death from the disease.

For current mental health patients, the American Psychiatric Association has already raised the alarm that the spread of COVID-19 can create barriers for access to psychiatric services.^73^ One prime example concerns patients on medication-assisted treatment (MAT) such as methadone and buprenorphine, who may face difficulty in physically attending their drug service or pharmacy at the frequency needed. In the UK, reports have emerged of pharmacies restricting access to MAT owing to reduced capacity, and patients stopping their treatment because of anxieties surrounding COVID-19.^74,75^ The implications for access to other medications that require frequent monitoring, such as clozapine, also need to be considered carefully. This is especially so when monitoring is indicated owing to the treatment's side-effect profile, which could also increase mental health patients’ vulnerability to COVID-19.^76^

In a recent survey by the Royal College of Psychiatrists (RCPsych), 43% of psychiatrists reported an increase in emergency cases, despite seeing a 45% decrease in their routine appointments.^77^ Professor Wendy Burns, president of RCPsych, stated:^77^

‘Our fear is that the lockdown is storing up problems which could then lead to a tsunami of referrals’.

As well as leading to increased incidence of mental health disorder, COVID-19 can also exacerbate existing conditions in current mental health patients and unmask existing symptoms in those without a current mental health diagnosis. Patients’ reluctance to seek help during the current pandemic, coupled with the reduced availability of routine appointments, could lead to a ‘tsunami of referrals’ post-lockdown – a situation that could easily overwhelm an overstretched and underfunded mental health service.^77,78^ This is further exacerbated by reduced provision for services deemed ‘non-essential’ in treating the acute medical problem, such as mental health services, in response to the outbreak.^59^ Without timely and adequate interventions, the compromised mental health system might not be able to cope with the potential surge in demand, as in Hong Kong during the SARS outbreak.^79^

## COVID-19 – the perfect vector

Anxiety, anger and stress are normal reactions to extremely adverse events such as the COVID-19 pandemic.^80^ For this reason, it is important that early mental healthcare intervention is provided to prevent progression into longer-term psychiatric conditions such as PTSD. The psychological needs of the population must be part of the public health response.^80^

As discussed, infected individuals are more likely to face severe psychological crises and secondary trauma after the disaster, a fact that must be taken into account when devising treatment strategies for COVID-19 patients. Efforts must be focused on identifying vulnerable populations, such as those with pre-existing mental health conditions, healthcare workers and families of affected individuals.^16^ Establishing key target groups during the initial stage of the outbreak, where the burden on services is significant and resources are scarce, allows for efficient and optimal use of limited resources.^81^ Providing precise and clear information regarding measures that enhance individuals’ perceived control over the threat may help engender coping methods that limit anxiety.^19,29,30^ Specific measures should also be taken to ensure that the psychological needs of quarantined or isolated individuals are accounted for.

Mental health services should brace themselves for a ‘mental health tsunami’^77^ in the months and potentially years to come, as the question of a secondary mental health epidemic is not a matter of whether it will happen, but rather to what extent will it happen. The concept of ‘flattening the curve’ in response to COVID-19 cases has been repeated by Prime Minister Boris Johnson on multiple occasions;^82^ similarly, steps should be taken to account for the mental health effects of COVID-19 as part of the curve which needs to be flattened, so as to not overwhelm our already overstretched mental health services.

## About the authors

**Idura N. Hisham** is a medical student at the Faculty of Medicine, St George's Hospital Medical School, London, UK. **Giles Townsend** is Consultant Old Age Psychiatrist at Surrey and Borders Partnership NHS Foundation Trust, The Meadows Inpatient Unit, Older Adults and Specialist Services, West Park, Epsom, UK. **Steve Gillard** is a Professor of Social & Community Mental Health at the Population Health Research Institute, St George's, University of London, UK. **Brishti Debnath** is a medical student at the Faculty of Medicine, St George's Hospital Medical School, London, UK. **Jacqueline Sin** is a NIHR Post Doctoral Research Fellow at the Population Health Research Institute, St George's, University of London, UK, and an Associate Professor in Clinical Health at the School of Psychology and Clinical Language Sciences, University of Reading, UK.

## Supplementary material

For supplementary material accompanying this paper visit http://dx.doi.org/10.1192/bjb.2020.60. click here to view supplementary material
